# The effect of impaired velocity signals on goal-directed eye and hand movements

**DOI:** 10.1038/s41598-023-40394-0

**Published:** 2023-08-22

**Authors:** Cristina de la Malla, Alexander Goettker

**Affiliations:** 1 Vision and Control of Action Group, Department of Cognition, Development, and Psychology of Education, Institute of Neurosciences, Universitat de Barcelona, Barcelona, Catalonia Spain; 2 Justus Liebig Universität Giessen, Giessen, Germany; 3 Center for Mind, Brain and Behavior, University of Marburg and Justus Liebig University, Giessen, Germany

**Keywords:** Human behaviour, Perception, Sensorimotor processing

## Abstract

Information about position and velocity is essential to predict where moving targets will be in the future, and to accurately move towards them. But how are the two signals combined over time to complete goal-directed movements? We show that when velocity information is impaired due to using second-order motion stimuli, saccades directed towards moving targets land at positions where targets were ~ 100 ms before saccade initiation, but hand movements are accurate. Importantly, the longer latencies of hand movements allow for additional time to process the sensory information available. When increasing the period of time one sees the moving target before making the saccade, saccades become accurate. In line with that, hand movements with short latencies show higher curvature, indicating corrections based on an update of incoming sensory information. These results suggest that movements are controlled by an independent and evolving combination of sensory information about the target’s position and velocity.

## Introduction

Moving objects normally capture our attention, which is revealed by the tendency of quickly directing our gaze towards them (e.g.^1,2^). When we attempt to interact with moving objects our oculomotor behavior is linked to the target’s movement (for recent reviews see^3,4^), unless we need to divert gaze from it towards other places or items we need or want to gather information from^5–14^. Looking at the moving target one aims for brings several advantages: targets are recognized better^15,16^ due to the highest visual acuity in the fovea compared to peripheral areas^17,18^, judgements of the target’s characteristics and predictions about how it will continue moving are more accurate^19–21^ and precise^22^, helps to direct and guide the hand towards targets of interest^23–27^, and errors in goal-directed actions caused by perceptual errors are reduced^6^. Moreover, proprioceptive information about how the eyes move during the pursuit phase^28,29^ and the efference copy that allows comparing the output of the ongoing and the planned movement^29–35^ can serve as extraretinal signals to judge the velocity at which targets move^36^.

Accurate tracking of moving targets is achieved by a combination of saccadic and pursuit eye movements, which despite their different characteristics and dynamics interact to reduce position and velocity errors (for reviews see^37,38^). Typically, to look at moving objects, we first direct our gaze towards them by making an initial saccade that will end with the retinal projection of the object of interest close to the fovea. As for other movements, predictions are needed^39,40^ to overcome inherent sensorimotor delays^41–43^. These predictions allow to bring gaze to the current target’s position rather than to the target’s position at the moment the movement was planned^44,45^. Therefore, for saccades to be accurate one needs information about the targets’ position and velocity (which will allow predicting where targets will be in the future), about the sensorimotor delays (which indicates the time needed to react to new sensory information) and about the time it will take to complete the saccade (which indicates towards which of the predicted positions the saccade must be directed to)^46^. Initial saccades are followed by smooth pursuit periods supported by slow rotations of the eyes (sometimes combined with small corrective saccades^47^) that allow keeping the moving target foveated throughout its trajectory.

Even though in physical terms velocity is just the change in position of an object over time, in the brain there seem to be different pathways to compute velocity signals. Targets encountered in the real world and commonly used in experimental designs differ in luminance from the background. Such differences in luminance over space and time can be measured by spatio-temporal filters to compute motion energy^48^. This mechanism is often called first-order motion and can be behaviorally and neurophysiologically dissociated from what is known as second-order motion (e.g.,^49–55^). Second-order motion targets are designed to not be detectable by spatio-temporal luminance filters (e.g., isoluminant chromatic targets or targets defined only by a moving texture). An important difference in how these two types of motions are processed is that second-order motion is mediated by detecting changes in position rather than directly computing velocity^50^. Moreover, second-order motion is substantially impaired in the periphery^56,57^, which also indicates that the processing of the velocity signal is affected. Yet, it has been shown that if there is enough time available, processing of second-order motion can lead to reliable information: while discrimination of motion direction for first-order motion stimuli is possible after seeing the stimuli for just a few milliseconds, second-order motion stimuli need to be presented for at least 200 ms^58–61^.

Disrupting the velocity-related information by using second-order motion targets has a direct impact on eye movement behavior. In a previous study^46^, participants had to follow two-dimensional step-ramp targets with their gaze as accurately as possible. Each trial started with participants looking at a starting point that appeared either at the top or the bottom of a computer screen placed in front of them. After a random delay, a target appeared in the center of the screen and started to immediately move horizontally. Importantly, targets could be either defined by luminance (first-order motion targets with high or low luminance contrast) or were isoluminant chromatic targets (second-order motion targets). Results showed that initial saccades directed to moving high- and low-contrast targets were quite precise. Such accuracy indicates that both position and velocity information were accurately taken into account, even when aiming at low luminance contrast targets with less reliable motion signals^56,62–64^. However, when saccades were directed towards second-order motion targets (i.e., the isoluminant targets) the landing position was approximately the position where the targets were 100 ms before the saccade started, which is the final information participants had available when they started to plan the saccade^65^. Interestingly, the impairment in processing the motion of isoluminant targets seems to be mainly present when the target moves in the periphery. When a second-order motion target is close to the fovea, saccades are accurate^46^ and pursuit is possible^19,66^.

The objective of this study is to extend previous findings by investigating how impairing the quality of incoming sensory information (by using second-order motion stimuli) influences isolated and coordinated eye and hand movements. As for eye movements, position- and velocity-related information is used to guide goal-directed hand movements^67–69^. This is evident from the fact that one corrects reaching movements if there are changes in either of these signals^70–75^. Investigating goal-directed hand movements towards second-order motion targets provides an interesting testbed. We expect information to be used in the same way for both isolated eye and hand movements. Therefore, impairing velocity information will have the same effect on both types of actions. Isolated saccades and hand movements should be accurate when aiming at first-order motion targets but end up behind the target by ~ 100 ms when aiming at second-order motion targets. However, the investigation of combined eye and hand movements may yield different outcomes. This is because initially executed saccades bring the eyes closer to the target location before the hand movement aimed at the same goal is completed, and as mentioned above, this proximity could offer additional information related to where the target is and how it moves. The impact of second-order motion targets on the accuracy of eye movements diminishes when the target is near the fovea^46^, and such an improvement in accuracy could also extend to more accurate hand movements. In addition, moving the eyes towards the target can further help to improve hand movement accuracy by directing and guiding it towards the target^23–27^. Consequently, when eye and hand movements are coordinated, we anticipate that the endpoints of hand movements will exhibit greater accuracy and remain unaffected by second-order motion stimuli. Alternatively, if initial saccades to second-order motion targets are only inaccurate due to the longer processing time that second-order motion targets require, the longer movement time for hand movements could allow to directly capture the effects of changes in the available sensory information.

## Results

**Figure 1:**
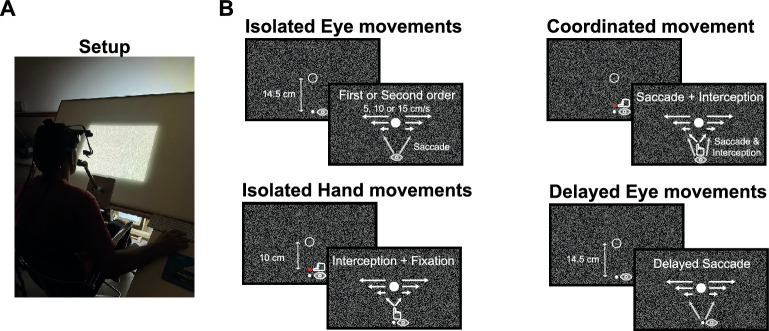
Setup and conditions. (**A**) Participants sat in front of a large screen onto which stimuli were projected from the back. To stabilize head movements, participants bite a bar with a dental imprint while performing the experiment. The bite bar was attached to the projection screen and had several degrees of freedom that allowed adjusting its position and distance to the screen so that participants were sitting in a comfortable way that allowed them to tap on the screen in the conditions requiring interception. We recorded eye movements using an Eyelink II. In some conditions participants started each trial by pressing the space bar of a keyboard located on their right side. In conditions requiring manual interception, we attached a marker on the index finger of the dominant hand that allowed a continuous recording of the hand trajectory. (**B**) Depiction of the different conditions. In the *Isolated Eye movements* condition, participants started fixating a position indicated in the lower part of the screen, and had to make a saccade towards the target. After the initial saccade, they had to try to follow the target with their gaze as accurately as possible. In the *Isolated Hand movements* condition, participants had to fixate throughout the trial at an indicated location in the lower part of the screen and try to intercept the targets by tapping on them. In the *Coordinated Eye and Hand movements* condition participants had to make a saccade towards the moving target and try to intercept it by tapping on it. In the *Delayed Eye movements* condition participants had to fixate their gaze in an indicated location in the lower part of the screen and hold the fixation until the fixation location disappeared. Please note that symbols in the figure are sometimes horizontally shifted for illustrative purposes. Background and fixation crosses were also modified for illustrative purposes.

To investigate how position- and velocity-related information are used and combined over time, we investigated goal-directed eye and hand movements towards first- and second-order motion targets. To test how information is used to perform different movements and to search for a temporal evolution of the relevant sensory signal (for a full description of the stimuli and procedure see the Methods section), we designed an experiment in which participants had to complete the following four different conditions (Fig. 1): (1) *Isolated Eye movements*: participants were required to make a saccade towards a target that moved across a large screen and to try to follow it with their gaze as accurately as possible after the initial saccade. (2) *Isolated Hand movements*: participants were required to fixate on a specific location on the screen and try to intercept a moving target by tapping on it without shifting their gaze towards the target. (3) *Coordinated Eye and Hand movements*: participants had to look at the target and try to intercept it at the same time. Here, bringing the eye to the target before the hand movement is complete could provide additional information about the target reflected in changes in the hand movements. (4) *Delayed Eye movements*: to assess the temporal evolution of the sensory signal in more detail, we had a condition where participants had to keep their gaze fixating in a specific location on the screen until that location disappeared. Once the initial fixation location disappeared, participants had to make a saccade towards a moving target, and follow it with their gaze as accurately as possible afterwards. The purpose of this final condition was to see whether additional processing time would change the accuracy of the initial saccade (see Methods for more details). In all conditions, the targets participants aimed for could randomly move leftwards or rightwards at either 5, 10 or 15 cm/s, and be defined by first- or second-order motion. The first three conditions were run in random order and counterbalanced across participants. The fourth condition was always the last one.

### Isolated Eye and Isolated Hand movements

**Figure 2:**
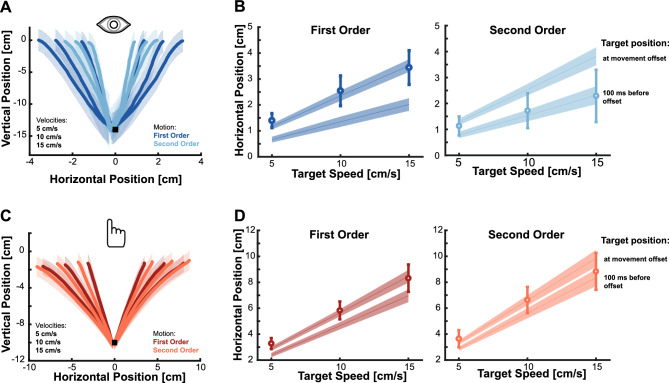
Isolated Eye and Isolated Hand movements. (**A**) Average movement trajectories of the initial saccade towards targets moving rightwards and leftwards at different velocities (5, 10 or 15 cm/s). Different shades of color indicate trajectories towards first- and second-order motion targets. (**B**) Circles show the average horizontal movement endpoint position for isolated eye movements when aiming at first-order (left panel) and second-order motion targets (right panel). Diagonal lines indicate the position of the target at movement offset (upper lines) and the position of the target 100 ms before movement offset (lower lines). (**C**) Same as in A, but for isolated hand movements. (**D**) Same as in (**C**), but for the isolated hand movements. Note that eye movements towards second-order motion targets land close to the position where the target was 100 ms before movement offset but hand movements are accurate. Shaded areas and error bars reflect the standard deviation across participants.

Figure 2 shows the average movement trajectories for isolated eye (Fig. 2A) and isolated hand (Fig. 2C) movements towards first- and second-order motion targets for the different target velocities. For isolated eye movements, the trajectories differ between first- and second-order motion. When looking at the endpoints, it becomes clear that saccades towards second-order motion targets land roughly 100 ms behind the actual position of the target while saccades directed to first-order motion targets are accurate (Fig. 2B). This reflects the impact of the impaired velocity signals on eye movements. In contrast (and against our initial hypothesis), the difference in hand movements aiming at first- and second-order motion targets is less obvious (Fig. 2D). Hand movements seem similarly accurate when aiming at first- and second-order motion targets.

**Figure 3:**
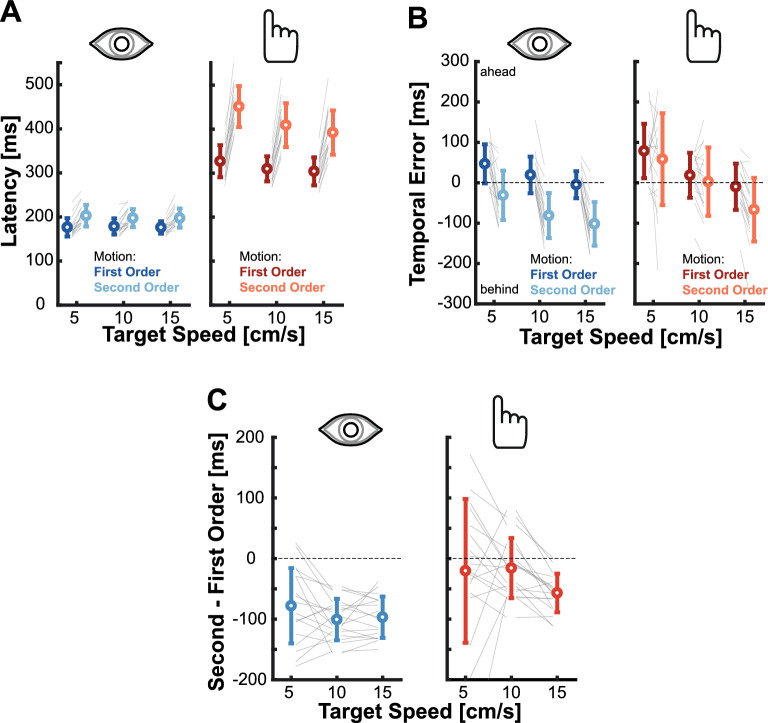
Behavior of isolated eye and isolated hand movements. (**A**) Average latency of eye (left panel) and hand (right panel) movements. Circles show the average for each target speed and target motion type, gray lines depict individual values. (**B**) Average temporal error for eye and hand movements. Same color-code as in (**A**). Positive values indicate tapping ahead of the target, negative values indicate tapping behind the target. (**C**) Relative temporal error between movements aiming at first- and second-order motion targets. Negative values indicate a lag for second-order motion targets with respect to the temporal error performed when aiming at first-order motion targets. Gray lines represent individual values, circles represent the mean across participants. All error bars reflect standard deviations.

To quantify differences in behavior, we computed the movements latencies as well as the temporal errors (see Methods for further details). For latency, a clear and expected pattern emerged. Latencies of both eye and hand movements were longer when aiming at second-order motion targets, and hand movements latencies were longer than eye movement latencies (Fig. 3A). For the critical metric of temporal error, we conducted a repeated measures ANOVA for the Isolated Eye and Isolated Hand movements conditions to examine the effects of the *motion type* (first- or second-order) and *target speed* (5, 10, or 15 cm/s). For the Isolated Eye movement condition we observed a significant effect of motion type (F_(1,17)_ = 105.259, p < 0.001), and of target speed (F_(1.445,24.565)_ = 78.608, p < 0.001), but no significant interaction (F_(1.385,23.550)_ = 2.854, p = 0.093). A similar pattern was present in the Isolated Hand movements condition. There was again a main effect of motion type (F_(1,17)_ = 5.520, p = 0.035) and target speed (F_(1.281, 21.776)_ = 41.555, p < 0.001) with no significant interaction (F_(1.175,19.971)_ = 2.388, p = 0.135). The main effect of target speed for both movements was driven by a more positive temporal error for the slowest target speed (5 cm/s), an intermediate value for the medium target speed (10 cm/s), and more negative temporal error for the fastest target speed (15 cm/s). This was especially prominent for the hand movements, where targets moving at a median speed had a temporal error close to zero, and the slower and faster targets were over- and underestimated, respectively (see Fig. 3B). This pattern of results suggests that the average target speed exerted an influence on participant’s movements and movement endpoints regressed towards the mean. We will further investigate this aspect later on, examining the role of average speed (refer to the subsequent section for more information).

The key factor for our hypotheses was the significant effect of motion type. Both isolated eye and isolated hand movements showed a more negative temporal error when aiming at second-order motion targets. However, a notable disparity was observed in the magnitude of this effect. To gain deeper insights into the influence of second-order motion, we calculated the difference in temporal error between first- and second-order motion targets for each target velocity (Fig. 3C). To directly compare the magnitude of the effect, we took those differences and performed a repeated measures ANOVA with *target speed* (5, 10 or 15 cm/s) and *effector* (Eye vs. Hand) as factors. There was a significant effect of the effector (F_(1,17)_ = 17.274, p < 0.001) but no significant effect of target speed (F_(1.183,20.112)_ = 3.787, p = 0.06) and no significant interaction (F_(1.236,21.005)_ = 1.612, p = 0.222). To take a closer look at the influence of the effector, we then calculated the average impact of second-order motion on eye and hand movements across target speeds. The results revealed a larger lag of -91.874 ms (SD: 37.993) for isolated eye movements compared to an average lag of -30.96 ms (SD: 57.510) for isolated hand movements. Based on our hypothesis, the impairment caused by second-order motion should introduce a 100 ms lag. To test for this, we conducted Bayesian one sample t-tests to examine whether the results were aligned with this expectation for both effectors. The results indicated strong evidence that the induced lag for hand movements was much smaller than our expected value (B_01_ = 0.003). Conversely, there was some evidence supporting the hypothesis that the observed effect for eye movements matched the expected lag (B_01_ = 2.866). Together, these results clearly demonstrate that the influence of the impaired velocity-related signal due to the use of second-order motion differs between eye and hand movements: while eye movements showed the expected lag of around 100 ms, isolated hand movements were less affected by second-order motion.

### Coordinated Eye and Hand movements

**Figure 4:**
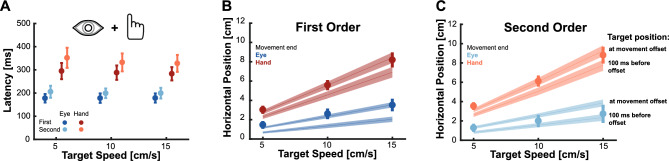
Behavior of coordinated eye and hand movements. (**A**) Average latencies for coordinated eye and hand movements. Circles show the average for each target speed separated by target motion type. The horizontal shift of the results for eye and hand movement is just for illustrative purposes. (**B**,**C**) Movement endpoints for eye and hand movements when aiming at first- (**B**) and second-order (**C**) motion targets. Shaded diagonal lines represent the target position at movement offset (upper lines) and 100 ms before movement offset (lower lines). Shaded areas and error bars represent standard deviations across participants.

Against our expectations, participants exhibited only slight impairment when intercepting second-order motion targets without looking directly at them. We initially hypothesized that hand movements could become accurate when accompanied by an eye movement as that would allow for a better analysis of the second order-motion target. However, the difference in the results obtained in the Isolated Eye and Isolated Hand movement conditions opens interesting additional possibilities for coordinated eye and hand movements: will the pattern of results be the same when executing coordinated rather than isolated eye and hand movements, or is there an interaction between the two movements that could help improve eye movements accuracy? Results show that indeed, the pattern of results is qualitatively comparable for isolated and combined movements. Movement latencies (Fig. 4A) were again longer for second- compared to first-order motion targets, and also clearly longer for hand than for eye movements. Regarding movement endpoints, we also observed a similar pattern to the one presented in the Isolated Eye and Isolated Hand movements conditions. When moving towards first-order motion targets, both eye and hand movements landed close to the actual position of the target (Fig. 4B). However, when aiming at second-order motion targets saccades still landed closer to the position where targets were 100 ms before movement offset but hand movements were accurate (Fig. 4C).

**Figure 5:**
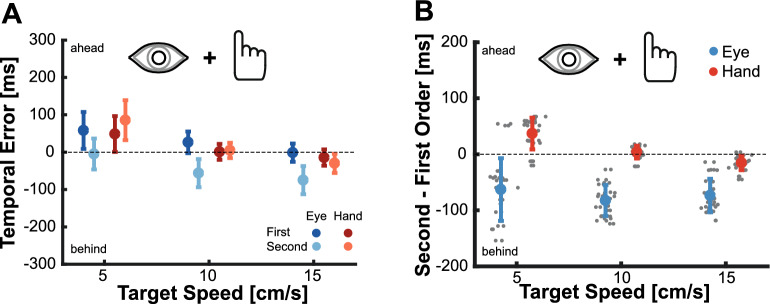
Effect of second-order motion targets on coordinated movements. (**A**) Average temporal error for the Coordinated Eye and Hand movements condition. Circles show the mean temporal error of the eye and the hand for each target speed separated by target motion type (darker and lighter colors). Please note that the horizontal shift of the results for eye and hand movement is just for illustrative purposes and that there are four data points related to the same target speed. (**B**) Relative temporal error between second- and first-order motion targets. Gray dots depict data from individual participants. Error bars show standard deviations across participants.

To test for the influence of *motion type* (first- and second-order) and *target speed* (5, 10, or 15 cm/s) on the temporal errors we again used a repeated measures ANOVAs now for the coordinated eye and coordinated hand movements. As for the isolated movements, the results showed a significant main effect of target speed for both eye (F_(1.116, 18.974)_ = 33.180, p < 0.001, no interaction (F_(1.312,22.301)_ = 1.747, p = 0.201) and hand movements (F_(1.032, 17.543)_ = 41.487, p < 0.001 and interaction F_(1.138, 19.342)_ = 36.675, p < 0.001) that again reflected a regression towards the average speed (Fig. 5A). However, the key question was again whether the coordination of eye and hand movements would change the impact of second-order motion on performance. There was a significant effect of motion type for both eye (F_(1,17)_ = 103.914, p < 0.001) and hand (F_(1,17)_ = 9.987, p = 0.006) movements. To look at this effect in more detail, we again computed the difference between the temporal errors when aiming at second- and at first-order motion targets (Fig. 5B). We repeated the same ANOVA as for the comparison of the two isolated movements with *effector* (Eye vs Hand) and *target speed* (5, 10 or 15 cm/s) as factors. The ANOVA showed significant main effects of the effector (F_(1,17)_ = 111.281, p < 0.001) and target speed (F_(1.303, 22.147)_ = 16.236, p < 0.001) and a significant interaction (F_(1.217, 20.688)_ = 5.740, p = 0.007). The main effect of target speed as well as the interaction were predominantly driven by the hand movements. In that case, the influence of second-order motion manifested as a positive difference for the slowest target speed, had almost no effect for the medium speed and resulted in a slight lag for the fastest target (Fig. 5B). When looking at the effect of the effector we observed that second-order motion led to an average lag of -72.939 ms (SD: 30.357) for the coordinated eye movements and an average lead of 8.589 ms (SD: 11.532) for the coordinated hand movements. Initially, our expectation was that in the coordinated condition, eye movements would still exhibit a substantial lag of 100 ms, while hand movements would be unaffected by second-order motion targets. To directly examine this hypothesis, we conducted Bayesian t-tests to compare the actual result with the expected lag of 100 ms. We found only little evidence for such a strong delay (B_01_ = 0.038). Similarly, for hand movements we tested the influence against zero (B_01_ = 0.119), but once again, we found insufficient evidence to support the absence of an effect of second-order motion. In fact, second-order motion did not produce a lag, but actually a significant lead.

### Comparison between isolated and coordinated movements

**Figure 6:**
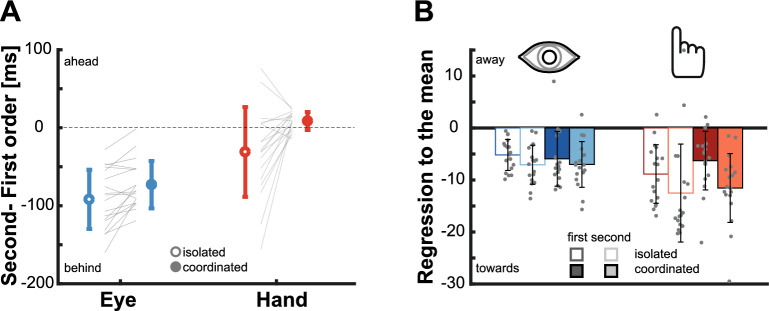
Difference between isolated and coordinated movements and the effect of the average speed. (**A**) The effect of second order motion is larger for the isolated than for the coordinated movements (the lag is reduced for both eye and hand movements). Gray lines show results from individual participants. Error bars represent standard deviations across participants. (**B**) We calculated whether participants relied on the average speed by fitting a regression line to the temporal errors observed across the different speeds. Empty bars show errors for the Isolated Eye and Isolated Hand movements conditions. Filled bars indicate saccades and hand movement errors in the coordinated movement condition. Negative values indicate reliance on the average speed and a related regression to the mean. Dots represent individual values, and error bars represent standard deviations across participants.

Initially we expected eye movements to land behind the target when aiming at second-order motion targets independently of whether the condition was the Isolated Eye movements or the Coordinated Eye and Hand movements. For hand movements we hypothesized that they would land behind the target in the Isolated Hand movements condition, but would be accurate in the Coordinated Eye and Hand movements condition as there would be a benefit of the target being foveally tracked by the time the hand reaches the screen. However, the pattern of results did not completely match our expectations. To directly examine whether the fact of simultaneously moving our eye and hand could alter motor control, we conducted a direct comparison between the estimated influence of second-order motion in the coordinated and isolated conditions (Fig. 6A). We observed that in the coordinated condition the lag was significantly reduced by about 30 ms for both eye (t_(17)_ = 3.662, p = 0.002) and hand movements (t_(17)_ = 3.108, p = 0.006). This improvement happened despite comparable latencies: the mean latency for eye movements towards second-order motion targets was 199.176 ms (SD: 21.677 ms) in the Isolated Eye movements condition and 201.331 (SD: 22.732 ms) in the Coordinated Eye and Hand movements condition. For hand movements the latency in the coordinated condition (M = 337.432, SD: 38.897 ms) was even much shorter than in the isolated condition (M = 417.550, SD: 47.500 ms). Hence, the enhancement in accuracy cannot be attributed to some speed-accuracy trade-off. Instead, these results suggest that performance of both eye and hand movements improves when executed in coordination with each other.

### The role of average speed

In addition to the effect of second-order motion, a consistent pattern we found across all conditions was the change in temporal errors from being more positive to being more negative depending on whether the target velocity was slower or faster, respectively. A potential explanation for such difference could be that participants relied on the average speed (10 cm/s) more than on the actual speed of the target. To quantify this effect, we fitted a linear regression to the temporal errors across the different target speeds for each condition. Here, a negative slope would indicate a regression towards the mean. Across all isolated and coordinated movements, almost all data points were negative, indicating a consistent inclination towards the average speed (Fig. 6B). To assess the magnitude of this effect across the different conditions, we conducted a repeated measures ANOVA with the factors *effector* (eye vs hand), *coordination* (isolated vs coordinated) and *motion type* (first- vs second-order motion). The results revealed that participants relied more on the average speed for second-order motion (F_(1,17)_ = 18.946, p = 0.035) than for first order-motion, and this reliance was more pronounced for hand than for eye movements ( F_(1,17)_ = 7.574, p = 0.014). There was no significant effect of coordination (F_(1,17)_ = 0.413, p = 0.529), and no systematic interaction between these factors (all p’s > 0.104). Together these findings point towards a more general rule, where there is a greater reliance on the average speed under more challenging sensory conditions. When targets were clearly seen (first-order motion) and obtaining information about how they move is easy, one can use the actual sensory information. However, as the target becomes more difficult to track (second-order motion), the influence of the average speed becomes more prominent, and this effect is stronger for hand than for eye movements.

### The relevance of processing time

**Figure 7:**
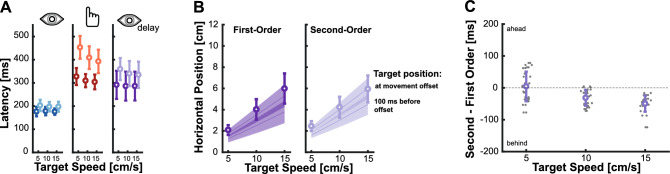
The role of processing time. (**A**) Latencies of the isolated eye (left), isolated hand (middle), and delayed eye movement (right) conditions. Different data points represent the mean for each target speed separated for the target motion type: first-order (darker color) and second-order (lighter color). (**B**) Average end position for the eye movements in the Delayed Eye movement condition for first-order (left) and second-order (right) motion targets. Saccades landing positions are shown as circles, reference target positions are represented by the shaded diagonal lines. Target position at movement offset is indicated by the upper lines, target position with a lag of 100 ms by the lower lines. **C** Relative temporal error between second- and first-order motion for the different target speeds. Gray dots depict individual data.

When examining isolated eye movements towards second-order motion targets, we observed the expected lag resulting from the impaired velocity signal. Interestingly, this lag was much less pronounced in isolated hand movements and was reduced for eye movements when performed in coordination with hand movements. Does this mean that second-order motion in the periphery is used differently for eye and hand movements (see e.g. ^76^.)? Or is this related to the temporal evolution of the available sensory signal with increased processing time? To assess this, we performed a critical comparison. Hand movement latencies were significantly longer than eye movement latencies (Fig. 7A). This difference allows for more time to process the second-order motion targets before executing and completing the hand movement unlike the rapid execution of a saccade. Therefore, rather than using information differently, it is plausible that after a longer exposure the motor system is capable of using an accurate estimate of second-order motion that may not yet be available at the moment of initiating a saccade. We tested this hypothesis by using the *Delayed Eye movement condition*, where we delayed the saccade onset by keeping the initial fixation cross on the screen for an additional 200 ms (see the Methods section for a full description of the experiment). This delay increased the time one is exposed to the target to values comparable to the ones observed in hand movements (Fig. 7A). Moreover, this is the time that has been suggested to allow processing of second-order motion stimuli^58–61^.

When we looked at the endpoints of the delayed saccades, we found an interesting effect: saccades towards both first- and second-order motion targets exhibit comparable accuracies (Fig. 7B). This behavior was quantified by computing the relative temporal error (Fig. 7C), which demonstrated a pattern resembling the results observed for hand movements. For the slowest target speed, there was no difference between second- and first-order motion targets, and differences were small for the other two target speeds. The average saccade lag for the delayed condition was -25.54 ms (SD: 25.609 ms). This lag was significantly smaller than the ones in the isolated (t_(17)_ = 7.730, p < 0.001) and coordinated (t_(17)_ = 7.416, p < 0.001) conditions. Hence, the increase in processing time resulting from the longer latencies appeared to contribute to the production of more accurate eye movements.

**Figure 8:**
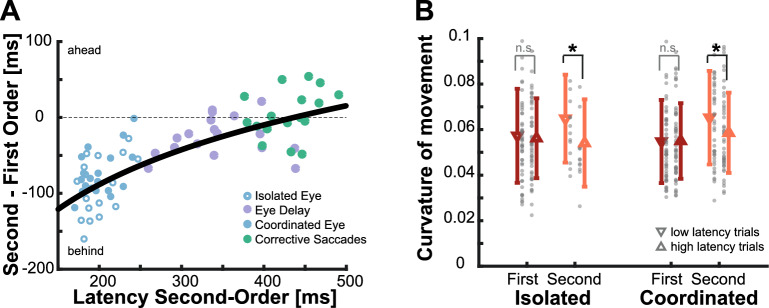
Temporal evolution of sensory signal. (**A**) The relative error between first and second-order motion for individual participants for the different conditions based on saccade latency to second-order motion targets. The black line depicts a logarithmic fit to the data. (**B**) Average hand movement curvature for conditions separated by low and high latency. The latency for the low group needed to be below 300 ms to qualify for this analysis. Averages are shown separately for first- and second-order motion targets (color) as well as isolated and coordinated movements (leftmost/rightmost symbols, respectively). Error bars depict the standard deviation across all qualifying data points (grey dots), see Methods for more details.

To look at whether there was indeed a signature of a temporal evolution of the available sensory signals, we computed the relative error between eye movements aiming at second- and at first-order motion targets, grouped by different saccade latencies for each of our conditions. When looking at Fig. 8A, one can notice that as saccades latencies increase, the impact of second-order motion diminishes, resulting in performance comparable to that of first-order motion targets when latencies reach approximately 300 ms or longer. In the analyses presented until now we mainly focused on the first saccade, but to gain deeper insights into the temporal evolution here we also analyzed the latency and performance of corrective saccades naturally occurring in the Isolated Eye movements experiment. While these corrective saccades exhibit varying amplitudes, they tend to occur after approximately 450 ms and show behavior consistent with the expected time course of evolving sensory information. This strongly suggests that processing velocity information about the movement of second-order motion targets requires more time than processing similar information for first-order motion targets. A typical initial saccade has a shorter latency and is thus initiated based on incomplete information. However, since hand movements are typically initiated later and allow for more time for online corrections, participants already have access to a more accurate representation of the target’s motion.

In a further exploratory analysis, we investigated whether we could observe a similar temporal evolution of the sensory signal revealed by hand movements. Since hand movements ended well after the critical time window of about 300 ms, we examined movement curvature and separated trials based on the hand movement latency. To ensure an adequate number of trials, we only included conditions where we had more than 10 trials for a participant with an average hand movement latency below 300 ms, which led to an uneven distribution of data points across conditions (see Methods for more details about the Analysis). We then compared the curvature for isolated and coordinated movements depending on movement latency. Short latency movements exhibited significantly higher curvature compared to long latency movements (Fig. 8B; isolated condition: t_(14)_ = 2.316, p = 0.036; coordinated condition: t_(42)_ = 2.460, p = 0.018). However, this effect was observed solely for second-order motion stimuli and not for first-order motion (isolated condition: t_(48)_ = 0.477, p = 0.636; coordinated condition: t_(51)_ = 0.113, p = 0.910). These findings provide some evidence that also hand movements with lower latencies show a signature of the temporal integration, as reflected in more pronounced trajectory corrections towards second-order motion targets.

## Discussion

The goal of this study was to investigate how impairing velocity-related information by using second-order motion influences isolated and coordinated goal-directed eye and hand movements. For isolated eye movements, we replicated findings from a previous study^46^, which demonstrated that initial saccades systematically landed behind the target by about 100 ms due to the weakened velocity signal (Fig. 2B). Surprisingly, isolated hand movements toward the same targets exhibited greater accuracy and roughly went to the right place at the right time (Fig. 2D). In addition, we observed that movement endpoints generally regressed towards the mean, especially when the target information was less reliable for the second-order motion targets (Fig. 6). When eye and hand movements were executed simultaneously, we found a similar pattern to that of isolated movements but a reduced effect of second-order motion: initial saccades still undershot targets, although the lag was reduced, and hand movements were even more accurate (Figs. 4 and 5). However, the striking difference between eye and hand movement control does not appear to be attributable to differences in sensory processing, but rather to movement latency. Over time, an accurate representation of the velocity of the second-order motion target seems to become available. Thus, when saccades were delayed to equal the duration of exposure to the second-order motion target with that of intercepting it with the hand, saccades became accurate (Figs. 7 and 8). Consistent with this, hand movements also exhibited a signature of an evolving sensory signal, as evidenced by the increased movement curvature in hand movements with shorter latencies, specifically for second-order motion targets (Fig. 8B). Overall, we observed two main factors modulating the influence of impaired velocity signals: the execution of coordinated eye and hand movements and the processing time.

### Control of goal-directed movements by a combination of position- and velocity-related information

Our results provide further evidence supporting the notion that movements towards dynamic targets seem to be controlled by an evolving combination of position and velocity information. Such proposals have been made for hand^67,69,77^ as well as for eye movements^78^; see also^37,38^. Our results suggest that the position-related information used for controlling a movement represents accurate, albeit delayed, information of the target, and that velocity-related information is integrated to compensate for this delay. When the velocity-related information is impaired (e.g., by using second-order motion), saccades land at the delayed position. The observed systematic temporal lag of around 100 ms (see Fig. 2B), makes it unlikely that the observed effect is just a larger error, as a consistent temporal delay requires errors to scale with target velocity. The errors we have found are consistent with using representations of the target’s position around 50–100 ms before the movement, and this is the typically considered critical window for the integration of sensory information for the movement plan (“saccadic dead time”, see for example^65^ for eye movements; and^69,77,79–81^ for interception models using similar delays). In addition, the systematic error of 100 ms behind the target suggests that the system has access to an accurate representation of the position of the target over time. However, and in contrast to our physical definition of velocity, the brain is not capable of directly computing velocity based on changes in position over time. Computing velocity of second order-motion targets seems to involve different processing mechanisms that require longer processing time (Fig. 8).

In our study, the impaired velocity-related signal was caused by the second-order motion stimulus. It is known that second-order motion, such as isoluminant chromatic targets, can attenuate velocity-related signals in area MT^82–87^ and affect speed perception^88,89^, for reviews see^56,90^. Despite this impairment, it is still possible to compute and use velocity information for second-order motion targets. Pursuit eye movements can be directed towards second-order motion stimuli defined by chromatic information^19^ or stimuli defined by different contexts, such as texture-based information^66,91^, although these circumstances may result in longer reaction times and affect initial pursuit acceleration. Furthermore, the position of the second-order stimuli seems to be critical. The position-based second-order motion system is attenuated in the periphery while pursuit can be initiated to targets located near the fovea (e.g.,^56,57^. The study of Goettker et al.^46^ indicated a behavioral reflection of this attenuation. While initial saccades and subsequent pursuit responses were accurate when directed towards isoluminant chromatic targets near the fovea, saccades towards targets moving in the periphery showed a systematic lag. In our study, we replicated this effect using texture-based second-order motion in the periphery. The attenuated velocity signal resulting from our stimulus and task elicited behavior similar to that observed in previous neurophysiological studies that showed impaired saccades to moving targets after an MT lesion^92^. Importantly, this observed effect points to a selective impairment of a velocity-related signal. Saccadic eye movements towards stationary targets were not affected by MT-lesions and also grasping of static isoluminant stimuli is preserved^93^. This selective impairment is also supported by the consistent movements towards a delayed position of the target (Fig. 2), which emphasizes the importance of the combination of a position- and a velocity-related signal for accurate movement towards a dynamic target.

### Coordination of hand and eye movements

Next to the relevant sensory information, one factor that influenced movement endpoints was how the movements were executed. When comparing isolated eye or hand movements with those being performed in a combined manner, we observed that both movements showed a higher accuracy and less influence of second-order motion. This effect was also independent of the influence of processing time as the average latency was comparable or even significantly faster for combined eye and hand movements.

An improvement of hand movement accuracy when performed in combination with an eye movement might be due to multiple potential effects. First, fixating on a different location than the aimed goal is quite unnatural (also visible in the large number of trials that needed to be excluded in that condition), since during regular everyday tasks gaze is used to locate and guide interactions with the environment^26^. Second, having the eyes closer to the target improves the acuity of incoming sensory information and tracking the target can improve the processing of moving targets^20,22^. Third, the gaze point can be used to anchor the hand movement^94^ and help to initially direct and then guide the hand towards the goal^23,24,70,72,95,96^. Fourth, and related to that, hand movements can not only use the final gaze position, but already efferent signals about upcoming eye movements to improve reach accuracy^33,34^. For example, even in the dark when pointing at a somatosensory target, an accompanying eye movement improves hand movement accuracy, although it does not provide of any new visual information^35^. While all these factors probably contributed to improve the hand’s movement accuracy when aiming at first-order motion targets, the reduction of the impairment of second-order motion targets despite shorter movement latencies is interesting. The successful use of gaze as an anchor or the integration of efferent information from eye movements requires an accurate eye movement, but for the coordinated movements the initial eye response still landed significantly behind the target. Therefore, we believe that the major source of benefit when manually aiming at second-order motion targets was a quicker and improved access to a velocity-related signal about the target, since the position-based second-order motion system works better closer to the fovea (e.g. ^56,57^,), where accurate interceptive movements are possible despite shorter latencies^78^.

However, a similar explanation does not hold for the differences between isolated and coordinated eye movements. The observed lag for eye movements behind the first-order motion target was reduced from around 100 ms to 80 ms, despite the movements having comparable latencies. These results could suggest that there is an influence of an efferent arm movement signal, although that would also mean that for hand movements a more accurate velocity-related signal becomes available earlier, which seems unlikely. The more likely explanation is probably that there are bidirectional benefits for coordinated movements that lead to an improved movement accuracy^35^, which is especially helpful for the uncertain situation of second-order motion.

### The integration of previous experience

One consistent pattern we observed in behavior was that movements were not only related to the current sensory information, but also influenced by the average target velocity. Specifically, we observed that endpoints for slow targets (5 cm/s) were usually ahead of the target, while movements towards fast targets (15 cm/s) ended behind the target. Movements towards targets moving at a medium speed (10 cm/s), which coincided with the average velocity exhibited higher accuracy. Such regressions to the mean have been previously reported in other tasks as well^97–99^. For example, Jazayeri and Shadlen^100^ demonstrated systematic regression to the mean in a task where participants had to reproduce time intervals. These effects have been attributed to the individual’s tendency to evaluate stimulus based on their relations to other stimuli and to the knowledge of one’s own temporal uncertainty. This internal estimate of uncertainty might also explain the difference we observed between eye and hand movements: The fast saccadic eye movements only have limited temporal uncertainty and limited time for potential influences of feedback and therefore only show a smaller regression effect than the longer and more flexible hand movements. Using a time-to-contact task, Chang and Jazayeri^101^ also showed that humans efficiently estimate prior statistics with information about speed and time to judge impending collisions. In line with these results, we also observed that the strength of the influence of previous experience depended on the reliability of the available sensory information. The effect was generally larger for the more challenging second-order motion stimuli (Fig. 6A). This suggests that observers relied more on their previous experience for target speeds with the most uncertain sensory information. Such a reliability-weighted integration of different types of information has proven successful in explaining behavior^102,103^. Recently, multiple studies have provided direct evidence for a reliability-weighted integration of previous experience and sensory information for the control of motor behavior even on a trial-by-trial basis^104–106^. Therefore, the observed influence of the average target speed seems to reflect a broader strategy of combining sensory information with previous experience, with greater reliance on the latter in the presence of uncertainty.

### Movements can serve as a continuous readout of evolving information

While isolated eye movements showed a systematic lag with respect to the target, we observed that isolated hand movements were quite accurate (Fig. 2D). One big difference between eye and hand movements was their latencies. Differences in latencies lead to differences in the amount of time one sees the target in the periphery prior to the movement. When controlling for the exposure time by delaying the eye movement response (*Delayed Eye movement* condition), saccades became more accurate and seem to incorporate an accurate velocity signal (Fig. 8). This suggests that although the computation of a velocity-related signal was initially impaired, with enough time it was possible to establish a reliable estimate. This is in line with psychophysical evidence that suggests that the correct identification of movement direction for a second-order motion stimulus indeed needs time: while discrimination of motion direction for first-order motion stimuli is possible already with very brief presentations, second-order motion stimuli need to be presented at least for 200 ms^58–61^. According to these findings, when we delay the saccade and increase the exposure time to the second-order motion signal we expected movements to be based on the updated signal and be more accurate. This time matches the observed hand movement latencies (Fig. 7A) as well as the time course of the reduction in lag with increased saccade latency (Fig. 8A). Saccadic eye movements here indeed showed comparable behavior to first-order motion targets with latencies starting around 250–300 ms. In addition, also additional corrective saccades, that happened later during the trial also showed no influence of second-order motion. A similar temporal evolution was also present for the hand movements. While on average even the isolated hand movements were accurate, when looking in more detail, we observed that hand movements with short latencies below 300 ms, had a significant larger curvature (Fig. 8B). This seems to be directly linked to corrections based on the evolving sensory information.

This temporal evolution in behavior raises two interesting points. First, movements can serve as a direct readout of evolving sensory information. The change in behavior with longer movement latencies seems to reflect the time course of processing of second-order motion. Previous work has already shown that the initial pursuit response reflects the dynamics of evolving signals in area MT^107^ or that responses to targets flashed during pursuit eye movements need time to become spatially accurate^108^. Eye movements can also serve as a window into decision making^109–111^; see also ^112^, or even reflect higher level cognitive factors such as surprise^113,114^, problem solving^115,116^ or deliberation^117^. Second, the motor system is not aware of the significant improvement that would come with longer latencies. If that would be the case, saccades could be simply always delayed and only triggered with all available information. However, since vision is an active process^118,119^ and continuous eye movements are needed to scan our environment, saccades are rather triggered regularly with incomplete information and do not integrate all available recent input^120,121^. The cost of a potentially inaccurate saccade which needs additional corrections seems to be outweighed by the improvement in resolution of a relevant object, even when the eyes do not land accurately on the target.

### Conclusion

We aimed at investigating the contributions of position- and velocity-related signals for goal-directed eye and hand movements over time. We observed that goal-directed movements were based on a readout of continuously evolving sensory information. When sensory information was less reliable, e.g., for isolated hand movements while fixating away from the target or when aiming at second-order motion targets, movement endpoints were shifted towards the average target velocity, suggesting a reliability-weighted integration of previous experience under uncertain conditions. For movements with short latencies accurate but delayed information about the target’s position was available, but the impaired velocity-related information prevented predictions of the future target’s positions and caused movements to systematically end behind the target. However, over time there was a successful integration of velocity-related information for second-order motion targets, which was reflected in a higher movement curvature for hand movements with latencies below 300 ms and more accurate eye movements when their onset was delayed or they naturally occurred during a later point in time.

## Limitations

While we found evidence for an evolving sensory signal underlying different goal-directed movements, one question we cannot answer with the current data is whether there is a continuous or categorical improvement of the velocity-related information. It could be the case that at some point the velocity-information is accurate and every movement that is based on this information is accurate, or that there is a continuous integration of an improved velocity-related signal over time. The individual variability of temporal errors between first- and second-order motion is quite large (Figs. 3 and 5). Therefore, we cannot really interpret the time course visible in Fig. 8A as a continuous temporal evolution of sensory behavior, since it could be influenced by the large individual variability between observers in our delay condition. To fully address this question, one probably would need to do a series of our delayed eye movement task with varying delays within each participant, to map the time course for each of them separately.

Furthermore, we initially expected that the isolated hand movements would show the same effect as the isolated eye movements. Since the combination of our experiments suggested that movements with latencies between around 300 ms already became quite accurate, we could only find an increased curvature denoting online corrections. Ideally however, we would also be able to show that hand movements could reveal the systematic lag behind the target offset, but for that we would either need hand movements with comparable latencies to saccadic eye movements or would need to somehow restrict the available processing times. The most likely solution would be to let observers intercept the targets, but occluding the second-order motion ones after varying periods of time.

## Methods

### Participants

One author and seventeen naïve participants took part in the study (age-range 19–44, 11 males, 1 left-handed). All participants except the author were naïve to the experiment purposes. All participants had normal or corrected-to-normal vision (with contact lenses), and none had evident motor abnormalities. Participants gave their written informed consent before taking part in the study, and were compensated with 50€ for their participation. The experiment was approved by the Ethics Committee of the University of Barcelona (Institutional Review Board 00003099), and was carried out in accordance with the approved guidelines.

### Apparatus

The experiment was conducted in a dark room. During the experiment, participants sat in front of a large screen (acrylic rear projection; width: 132.5, height: 84.5; tilted backwards by 40º) onto which stimuli were projected (Casio Laser Led Projector; resolution 1024 by 768, screen refresh rate: 120 Hz) with a delay of 44 ms (all delays were accounted for during the analyses).

We recorded both eyes’ movements with an Eyelink II (SR Research) at 500 Hz. We used a biteboard with a dental imprint to fix the participants heads’ position. The biteboard was located at the extreme of a custom-build structure attached to the bottom part of the large screen (Fig. 1A). This structure had different degrees of freedom, which allowed participants to adjust its orientation and place their mouth on the biteboard comfortably while either just looking at the screen or when having to move their hand to try to intercept the moving targets by tapping on them. The distance between the participants’ eyes and the screen was approximately 55 cm. To be able to correctly calibrate and record eye movements, we limited the projection area to be a 54.5 by 40.5 cm rectangle. Where participants were looking on the screen was determined following a standard 9 points calibration prior to starting each block of trials. Only when the validation error was < 0.35 deg for both eyes the calibration was considered successful. Otherwise, the calibration procedure was repeated.

To record hand movements in the interception tasks we used a Polhemus Liberty electromagnetic tracker (Polhemus, 40 Hercules Drive, Colchester, Vermont). We attached a motion sensor to the index fingertip of the participant’s dominant hand to record hand movements at 120 Hz. The motion tracker was calibrated prior to each interception block. For the calibration we measured the position of the sensor when it was placed at 30 indicated positions on the screen. At the end of the calibration, a white dot was drawn showing the position of the sensor to check whether the calibration was correct. We checked whether the calibration was correct by looking at whether the dot remained aligned with the index finger when participants placed their finger in different positions of the screen. If this was not the case, the calibration procedure was repeated.

### Stimulus and procedure

We used second-order motion targets to look at the effect of impairing velocity information in saccades and hand goal-directed movements, and to understand how position and velocity information is used in different cases. Participants completed four conditions that followed a similar design to that previously used by Goettker et al.^46^. The first three conditions were completed in random order and counterbalanced across participants. The fourth condition was always completed at the end.

### Isolated Eye movements condition

Participants started each trial by fixating a stimulus presented 14.5 cm below the screen center (Fig. 1B). The stimulus participants had to fixate consisted of a combination of a circle and a cross to try to ease a stable fixation^122^. The background consisted of a contrast-defined (black and white) random dot texture^50,123^ that changed every 80 ms. Each texture element was 0.05 by 0.05 cm. After a random time between 1 and 1.5 s, the fixation location disappeared and a target appeared in the center of the screen and immediately started moving to the left or to the right. Targets could be either a 1 cm diameter white mask (first-order motion targets), or a 1 cm diameter mask composed of the same texture elements as the background and updated with the same frequency (second-order motion targets). In this last case, the target does not differ from the background in luminance and one can see it while it moves, but not when it is static (a video of the stimulus can be seen following this link https://osf.io/w9hpz/). Notice also that even though the change in the background and the target texture occurred with the same frequency (every 80 ms) the fact that the stimulus moves in one direction makes it possible to distinguish it from the background. Participants were instructed to look at the moving target and then follow the target with their eyes as accurately as possible. Targets could move at either 5, 10 or 15 cm/s, and for either 650, 700 or 750 ms. Once the target disappeared, participants had to press the space bar on a computer keyboard placed next to them (Fig. 1A) and fixate at the bottom of the screen to start the following trial. Each participant performed 4 blocks of randomly interleaved trials (2 types of targets × 2 directions × 3 velocities × 3 presentation times × 5 repetitions of each combination, 180 trials in total). Each block lasted approximately 15 min. Participants could have a break anytime they wanted by not pressing the space bar. With this condition, we expect to replicate the results previously reported by Goettker et al.^46^. We expect saccades directed towards first-order motion targets to be accurate, and saccades directed to second-order motion targets to land at positions where targets were ~ 100 ms before the saccade started. This would confirm that the effects found when aiming at isoluminant targets extend to other second-order motion targets.

### Isolated Hand movements condition

In this condition participants saw the same stimuli as in the previously described condition. The only difference is that a 1 cm diameter red circle was drawn 10 cm below the screen center (slightly above the initial fixation location, Fig. 1B). This circle indicated where participants had to place their index finger to start each trial. Between 1 and 1.5 s after participants placed their finger in the starting position the target appeared in the center of the screen and started moving either leftwards or rightwards. Participants were instructed to keep fixating on the initial fixation location while trying to intercept the target by tapping on it. To start the following trial, participants had to place the index finger back in the starting position. The number of trials per block was again 180. Each participant completed 4 blocks, each of them lasting for about 15 min. Participants could have a break anytime they wanted by not placing the index finger in the starting position. Our specific prediction in this condition is that if information is used in the same way for eye and hand movements, the same errors found in saccades will be found for interceptive hand movements. Therefore, we expect accurate interception when aiming at first-order motion targets and errors of ~ 100 ms when aiming at second-order motion targets.

### Coordinated Eye and Hand movements condition

In this condition participants saw the same stimuli as in the previous ones. Participants started each trial fixating at the initial fixation point and placing their index finger within the red circle (Fig. 1B). Between 1 and 1.5 s after participants placed their finger in the starting position the initial fixation dot disappeared and the target appeared and started moving. In this case, participants were instructed to follow the target with their eyes as accurately as possible, and to try to intercept it by tapping on it. As in the isolated hand movement condition, participants had to place the index finger back in the starting position to start the next trial. The number of trials per block was again 180. Each participant completed 4 blocks, each of them lasting for about 15 min. Participants could have a break anytime they wanted by not placing the index finger in the starting position. In this condition we have different predictions for the saccades and hand movements errors. Saccades are expected to be accurate when aiming at first-order motion targets but not when aiming at second-order motion targets (as in the isolated eye movement condition). However, as both the latency and the movement time of the hand are longer than that of the eye, we expect gaze to already be close to the target while the hand is still moving towards the target. Therefore, it would be possible to guide the ongoing hand movement towards the correct location of the target independently of whether it is a first- or a second-order motion target.

### Delayed Eye movement condition

Since saccade latencies are generally shorter than hand latencies (see the Results section) potential differences in the accuracy of eye and hand movements could indicate that position and velocity information are used differently for the two acting systems, or that the amount of time available to integrate visual information influences performance. To disentangle these two options participants completed a fourth condition once they had finished the three main ones. Stimuli were the same as in the Isolated Eye movement condition. The target appeared after a random period between 1 and 1.5 s after the trial started. Unlike in the Isolated Eye movement condition though, in this one we displayed the initial fixation dot for an additional 200 ms. Participants were instructed to delay their eye movement towards the target until the fixation dot disappeared. By doing so, the time participants were exposed to the stimuli before making a saccade towards it was similar to the time it took them to start moving their hand to intercept the moving target in the conditions involving interception. The number of trials per block was 180. Each participant completed 2 blocks, each of them lasting for about 15 min. Participants could have a break anytime they wanted by not pressing the space bar that started the following trial. Differences between eye and hand movements accuracy when their latencies are similar would indicate that position and velocity information are used differently for the two acting systems. Finding no differences when latencies are similar would indicate that the time during which one sees the target influences movement accuracy.

## Data analyses

### Pre-processing

The Eyelink and the Polhemus systems ran at different frequencies (500 vs 120 Hz), so to align both signals in time we use linear interpolation to obtain the hand position every 2 ms and be able to match both recordings.

We averaged the data from both eyes to estimate the gaze position during the trial. The distance between the participants’ eyes and the screen was not always exactly the same because participants were free to adjust the height of the chair, and to place the biteboard in a position so that they could sit, keep the biteboard on their mouth and reach the screen comfortably. For this reason, we report gaze velocity in cm/s on the screen, rather than on deg/s. For the analysis, we collapsed trials moving to the left and to the right, by multiplying the horizontal component of movements to the left by -1.

Gaze positions were taken from the Eyelink raw data and transformed into positions on the screen. Blinks were linearly interpolated. Saccadic eye movements were labelled from the Eyelink (velocity > 30 deg/s and acceleration > 4000 deg/s^2^). For each trial where we analyzed eye movement responses, we determined the latency of the first saccade after the target movement onset. Since targets moved horizontally and the correction for velocity errors was therefore along this axis, we calculated the horizontal saccade landing position. As a comparison, we also looked up the horizontal target position at the moment the saccade ended. As we expect saccades to land at past positions of the targets when aiming at second-order motion targets, we also calculated the horizontal position where the target was 100 ms before saccade offset.

We calculated the hand’s 2D velocity and then filtered it by averaging the velocity trace in a sliding window of 10 samples. We defined the hand movement onset as the first moment in time the hand reached a velocity larger than 10 cm/s and sustained it for at least 25 ms once the target had started to move. Hand movement offset was defined as the first moment in time the velocity of the hand was below the same threshold and stayed there for at least 25 ms. As for saccades, we calculated the horizontal endpoint of the manual interceptive movement and compared it to the target position at the moment of the tap and to the target position 100 ms prior to the hand movement offset.

### Metrics and statistical analysis

On each trial, we calculated the final temporal error participants made in their eye and hand movements with respect to the target. To do so, we divided the spatial error between the eye or the hand’s final position and the target position at that moment by the speed of the target on that trial. In this way, a negative value indicate that the movement ended behind the target, and a positive error that it ended ahead of the target.

For the Isolated Eye and Isolated Hand movements conditions, we hypothesized that while movements towards first-order motion targets should be accurate, movement towards second-order motion targets should land at positions where the targets were 100 ms before the movement, since this is the estimated time it takes to update a movement based on new position information^74,75^. To test the effect of motion type (first- or second-order) and target speed (5, 10 or 15 cm/s) on the temporal error we run a repeated measures ANOVA with these variables as factors for the Isolated Eye and the Isolated Hand movements condition. As our main interest is to look at the effect that second-order motion targets have on goal-directed eye and hand movements, we calculated the difference in temporal errors when aiming at second- or first-order motion targets for each target velocity. To explore this effect further and to directly compare it between the effectors, we ran a repeated measures ANOVA based on the differences to compare the magnitude of the effect of second order motion targets on performance. In this ANOVA we included the target speed (5, 10 or 15 cm/s) and the effector (hand or eye) as variables. Since we had specific effects about the magnitude of the influence of second-order motion, we averaged the differences across all target speeds and used Bayesian one-sample t-tests to check whether results match our expectations^124^. With this procedure we could explicitly assess the evidence for our results being not different than our initial expectations that for the isolated movements both hand and eye should show a lag of 100 ms. For the ANOVAs, we corrected for violations of sphericity and reported the Greenhouse–Geisser corrected degreed of freedom and p-values.

For the Coordinated Eye and Hand movement condition, we followed the same procedure as for isolated eye and hand movements. We initially assessed the influence of motion type (first- or second-order) and target speed (5, 10 or 15 cm/s) on the temporal error by running separate repeated measures ANOVAs for eye and hand movements. Again, to explore the effect of motion type further, we computed the difference in temporal error between second- and first-order motion and then ran a second repeated measures ANOVA to compare the effect across target speed (5, 10 or 15 cm/s) and effector (hand or eye). We then directly tested our initial expectations again by assessing the evidence for the eye movement showing a lag of 100 ms and the hand movements showing no influence by Bayesian one-sample t-tests.

To examine whether making a coordinated movement had some effect on the errors participants made we run paired t-tests to compare the average lag estimated based on the difference between the temporal error for second- and first-order motion across target speeds. We compared this influence of second-order motion for eye movements between the Isolated Eye movements condition and in the Coordinated Eye and Hand movements condition. The same tests were run to compare the lag when trying to intercept the target with their hand (i.e., between the Isolated Hand movement condition and the Coordinated Eye and Hand condition).

Since the interception task was quite demanding due to the short presentation times, and we observed consistent influences of target speed, we also analyze how much participants relied on information accumulated from previous trials rather than on the current sensory information. To quantify this, we fitted a linear regression to the temporal errors across target speeds for each condition for isolated and coordinated eye and hand movements. The slope of this regression indicates the direction and the strength of the regression to the mean, with more negative values reflecting a stronger influence of the average speed. We then compared the strength of the regression to the mean by using a repeated measures ANOVA with the factors effector (eye or hand), coordination (isolated or coordinated movements) and motion type (first- or second-order).

Due to the difference in latency between hand and eye movements, we were interested in how the difference in sensory information between first- and second-order motion targets changed with latency. For eye movements, we computed the relative timing error between second- and first-order motion targets and plotted it with respect to average saccade latency for second-order motion targets. We used data for each participant in the Isolated Eye, Coordinated Eye and Hand, and the Delayed saccade task. Additionally, we also added the same measurement for corrective saccades that occurred in the Isolated Eye movements condition.

To assess the temporal evolution for hand movements, we did an exploratory analysis. Interception endpoints typically happened after more than 500 ms, which is far outside of the critical time window below 300 ms. Therefore, we did not consider the endpoint, but looked into movement curvature. Curvature was defined as the maximum distance of the trajectory to a straight line fitted between the starting and endpoint of the movement divided by the length of this line. To estimate the effect of latency we compared the curvature between the 25% of trials with the lowest latency and the 25% of trials with the highest latency. We did so separately for each target speed, motion type (first- and second-order) and type of movement (isolated or coordinated condition). Since hand movement typically had long latencies, we only took data points into account where we had more than 10 valid trials and the fast latency group of trials had an average latency below 300 ms to match the critical time window observed for the time course of saccadic eye movements. We then used paired t-test to compare the curvature between the two latency groups for isolated and coordinated movements separately for first and second-order motion. The groups can have different number of trials, since especially for the isolated hand movements to second order motion trials there are only a few participants and conditions where the average of the lower latency group was below 300 ms. While target speed had an influence on curvature, please note that only pairs of data points with high and low latency for the same target speed were included, and the differences therefore cannot be explained by target speed.

### Exclusion criteria

Gaze data were excluded from the analysis when more than 500 ms of data was missing during the trial (227 trials for Isolated Eye, 159 trials for Coordinated Eye, 172 trials for Delayed Eye), if the latency of the saccade was shorter than 100 ms (1078 trials for Isolated Eye, 980 trials for Coordinated Eye, 307 trials for Delayed Eye), or if the saccade did not go towards the target. We defined saccades as not aiming to the target as saccades ending more than 5 cm below the target’s path or ending 500 ms ahead or behind the target position at landing time (1219 trials for Isolated Eye, 1034 trials for Coordinated Eye, 767 for Delayed Eye). Hand movement data were excluded from the analysis when the latency of the hand movement was shorter than 100 ms (67 trials for Isolated Hand, 37 trials for Coordinated Hand), or if we could not determine a valid movement onset or offset, based on the velocity criteria mentioned above or an endpoint still vertically more than 5 cm below the target, or not within 200 ms of the moment the target disappeared (703 trials for Isolated Hand; 180 trials for Coordinated Hand). Similar to eye movements, hand movements were also excluded when ending more than 5 cm below the target’s path or ending 500 ms ahead or behind the target position at landing time (414 trials for Isolated Hand, 263 trials for Coordinated Hand). In the Isolated Hand movements condition, trials were also excluded if gaze deviated by more than 5 cm from the fixation point (2850 trials). Across all conditions we included 34,734 of the 45,360 trials (77%) for the analysis. Exclusion rate slightly differed across the different conditions with 19.4%, 20.4% and 18.8% of excluded trials for the isolated eye, the coordinated movements, and delayed eye movement condition respectively, but 32.8% for the Isolated Hand movement condition. Please note here that valid trials for the Coordinated Eye and Hand movements condition were trials where eye and hand movements were determined as valid, the number of trials given above however are given per movement. Also note that despite the relatively high exclusion rate, due to the large number of total trials the minimum number of valid trials per condition (combination of target speed and motion type) per participant were 55 trials (mean = 96 trials) for the Isolated Eye movements condition, 15 trials (mean = 81 trials) for the Isolated Hand movements condition, 33 trials (mean = 95 trials) for the Coordinated Eye and Hand movements condition and 25 trials (mean = 48 trials) for the Delayed Eye movement condition.

## Data Availability

All data is available following this link: https://osf.io/w9hpz/. We also provide of a video showing how the stimuli we used in the experiment looked like.
